# Automaticity and Flexibility of S–R Retrieval During Priming

**DOI:** 10.3390/brainsci7060065

**Published:** 2017-06-13

**Authors:** Hope Tobin, Elizabeth Race

**Affiliations:** Department of Psychology, Tufts University, Medford, MA 02155, USA; elizabeth.race@tufts.edu

**Keywords:** repetition priming, associative learning, memory, context

## Abstract

Learned associations between stimuli and responses (S–R associations) make important contributions to behavioral and neural priming. The current study investigated the automaticity and flexibility of these S–R associations and whether the global task context in which they occur modulates the impact of S–R retrieval on priming. Participants engaged in a semantic repetition priming task in which S–R retrieval is known to influence priming. Across participants, repetition priming occurred in global task contexts (i.e., combination of activated task sets) that either remained consistent or shifted across time. In the stable context group, the global task context at study matched that at test, whereas in the shifting context group, the global task context at study differed from that at test. Results revealed that the stability of the global task context did not affect the magnitude of S–R contributions to priming and that S–R contributions to priming were significant in both the stable and shifting context groups. These results highlight the robustness of S–R contributions to priming and indicate that S–R associations can flexibly transfer across changes in higher-level task states.

## 1. Introduction

Priming is a powerful example of repetition-related learning in which repeated exposure to a stimulus facilitates subsequent behavioral and neural processing. Repetition priming has traditionally been thought to emerge from long-term learning at the stimulus level and changes in cortical representations of stimulus-specific conceptual or perceptual features [1,2,3]. Accumulating behavioral and neural evidence indicates that priming can also reflect learned associations between stimuli and responses (S–R associations), particularly during speeded classification tasks [4,5,6,7,8,9] (for review see [10]). S–R associations form when a response is repeatedly executed in the presence of a stimulus. For example, when one repeatedly makes a yes/no response about whether an object (e.g., “Knife”) is dangerous (e.g., “Yes”), an S–R association (i.e., “Knife-Yes”) is formed. Subsequent presentations of a stimulus can trigger the rapid retrieval of this learned S–R association. In priming paradigms, this S–R retrieval has been shown to facilitate decision making and conserve computational resources, as evidenced by faster, more accurate responding and reductions in neural activity when responses are repeated across stimulus presentations.

While S–R contributions to repetition priming are now widely recognized, important questions remain about the automaticity and flexibility of S–R retrieval. Of particular interest is the degree to which S–R associations transfer across shifts in task context [10]. One type of task context is the current task set (or activated decision rule) that determines the relevance of learned S–R associations [11]. Prior research has established that S–R retrieval occurs even when the task set (or decision) associated with a particular stimulus changes across stimulus repetitions [8,9]. A second, higher-level task context that may influence S–R retrieval is the combination of tasks sets that are activated and maintained over time when performing a cognitive task [12,13]. Task sets can remain stable or can shift over time depending on one’s goals or task instructions. Stable task sets occur when the same cognitive configurations remain active and relevant across time (e.g., when task instructions remain constant across blocks of trials), whereas a shift in task set occurs if the activated cognitive configurations change (e.g., when task instructions shift from one block of trials to the next block of trials).

Evidence from prior priming studies suggests that S–R retrieval may be robust to shifts in such higher-level task states. For example, Horner and Henson (2009) found that S–R associations learned in one task context (e.g., a block of “smaller than a shoebox” classifications) are automatically retrieved and influence behavior when subsequently encountered in a different task context (e.g., a block of “man-made” classifications). This finding suggests that S–R associations can transfer across shifts in global task context and that stable, higher-level task states may not be necessary for the retrieval of S–R associations during priming. However, it is also well established that memory retrieval improves when global environmental contexts remain constant across encoding and retrieval [14]. Recent research by Song and colleagues demonstrated that context-dependent retrieval extends to consistent dual-task contexts [15,16]. Specifically, Song and colleagues found that the concurrent performance of two tasks forms a long-term dual-task context that becomes integrated with visuomotor memory and that visuomotor memory improves when this dual-task context is reinstated at retrieval, even though dual-task contexts are typically more challenging than single-task contexts [15]. These results suggest that global features of cognitive sets (single-task vs. dual-task contexts) can become bound to visuomotor memories at encoding and that the reinstatement of these contexts at test can facilitate memory retrieval.

Waszak and colleagues (2013) also found evidence that the retrieval of S–R associations may be context-dependent and that automatic S–R retrieval can be modulated by top-down control states [17]. In this study, participants performed a target detection task in which S–R retrieval could be triggered by irrelevant distractor stimuli. The researchers found that distractor-based S–R retrieval only influenced target detection times when the S–R associations were consistent with the currently valid task set (i.e., the instructed S–R mapping). When the distractor-based S–R associations were not consistent with the currently valid task set, they did not influence target detection times. These results indicate that the automatic retrieval of S–R associations can be inhibited by instructions about relevant S–R mappings (e.g., task context), at least in the context of target detection tasks. Together with evidence that higher-level task contexts can influence the magnitude of behavioral facilitation in priming tasks [18], these results suggest that the automatic retrieval of S–R associations during priming may be modulated by top-down factors related to global task states.

Although S–R contributions to priming have been separately identified in both stable (e.g., [8,19]) and shifting (e.g., [9]) global task contexts, no study to date has directly tested whether S–R contributions to priming are reduced in shifting compared to stable task contexts. The current study investigated this question by having participants engage in a semantic repetition priming task in which they repeatedly classified stimuli (e.g., “Lion”) according to one of two classification rules (“Is the object smaller than a shoebox?” or “Is the object natural?”). Prior research has established that robust S–R associations are formed in this task and contribute to later behavioral and neural priming at test [8,19]. Importantly, we manipulated the stability of the global task context across participants such that it either remained consistent across study and test blocks (stable context group) or shifted between study and test blocks (shifting context group). If the S–R priming effect is smaller when contexts shift, this would provide evidence that higher-level features of the global task context can become bound to S–R representations and influence their subsequent retrieval. Such a result would align with the notion that shifts in ongoing task states can serve as an event boundary that impairs the transfer of S–R learning and reduces the impact of learned responses on behavior [20]. Alternatively, if the magnitude of S–R contributions to priming does not differ between stable and shifting task contexts, this would indicate that S–R associations can be dissociated from the global contextual features in which they were formed.

## 2. Materials and Methods

### 2.1. Participants

Forty individuals served as participants. Data from two additional participants were collected but excluded due to poor performance (<30% accuracy in one or more conditions). All participants were either native English speakers or individuals who learned English as a second language early in childhood. The experiment was arranged as a between-subjects design, with half of the participants assigned to the stable context group (8 male, 12 female; mean age = 20.6 years; mean education = 14.3 years) and half assigned to the shifting context group (4 male, 16 female; mean age = 20 years; mean education = 13.6 years). Participants were recruited from the Tufts University student body and the surrounding area and received either psychology course credit or $10/h for their participation. Before beginning the experiment, all participants gave their informed consent for inclusion. The study was conducted in accordance with the Declaration of Helsinki, and the protocol was approved by the Institutional Review Board of Tufts University (Project Code 1510002; 16 October 2015 approval).

### 2.2. Stimuli

The stimulus set consisted of 320 concrete nouns. Half of these nouns referred to objects smaller than a shoebox and half referred to objects larger than a shoebox. Similarly, half of these nouns referred to naturally occurring objects and half referred to man-made objects. During the study phase, participants viewed 128 of the nouns (primed stimuli) three times, intermixed with 128 novel stimuli. During the test phase, participants viewed the 128 primed stimuli again, intermixed with 64 new stimuli (Novel trials). Of the 128 primed stimuli shown at test, 64 occurred with the same task pairing as at study (Within-Task trials) and 64 were presented with the alternate task pairing (Across-Task trials). Novel, Within-Task, and Across-Task trials contained an equal number of nouns from each of the smaller/larger and natural/man-made crossings and all conditions were matched for mean word length and frequency. The stimuli were also counterbalanced across conditions and participants.

### 2.3. Procedure

The experiment was programmed in PsychoPy v.1.83.04, run on Mac OS X Yosemite v.10.10.5 (Apple Inc., Cupertino, CA, USA), and took participants approximately 45 min to complete. The experiment consisted of one 512-trial study block and one 192-trial test block. Reaction time and accuracy data were collected during both the study and test blocks, with analyses focusing on data from the test block. Before beginning the experiment, participants received verbal instructions and completed a practice block. Throughout the experiment, participants were encouraged to respond as quickly and accurately as possible.

The same trial structure was maintained across study and test blocks. At the start of each trial, participants saw a fixation cross appear at the center of the computer screen. Five hundred milliseconds later, a task cue (“SMALLER?” or “NATURAL?”) appeared above this fixation cross, indicating the classification decision to be made on that particular trial. The task cue “SMALLER?” prompted participants to decide whether the target noun was smaller than a shoebox (smaller decision), while the task cue “NATURAL?” prompted participants to decide whether the target noun occurred naturally in the environment (natural decision). Five hundred milliseconds after the task cue was presented, the target noun appeared below the fixation cross. At this point, participants made a classification decision about the target noun by pressing keys for “Yes” or “No” on the computer keyboard (“Yes” and “No” keys and fingers used to make the responses remained consistent across study and test phases). Once a response was recorded, the fixation cross, task cue, and target noun disappeared, and the next trial began.

**Figure 1:**
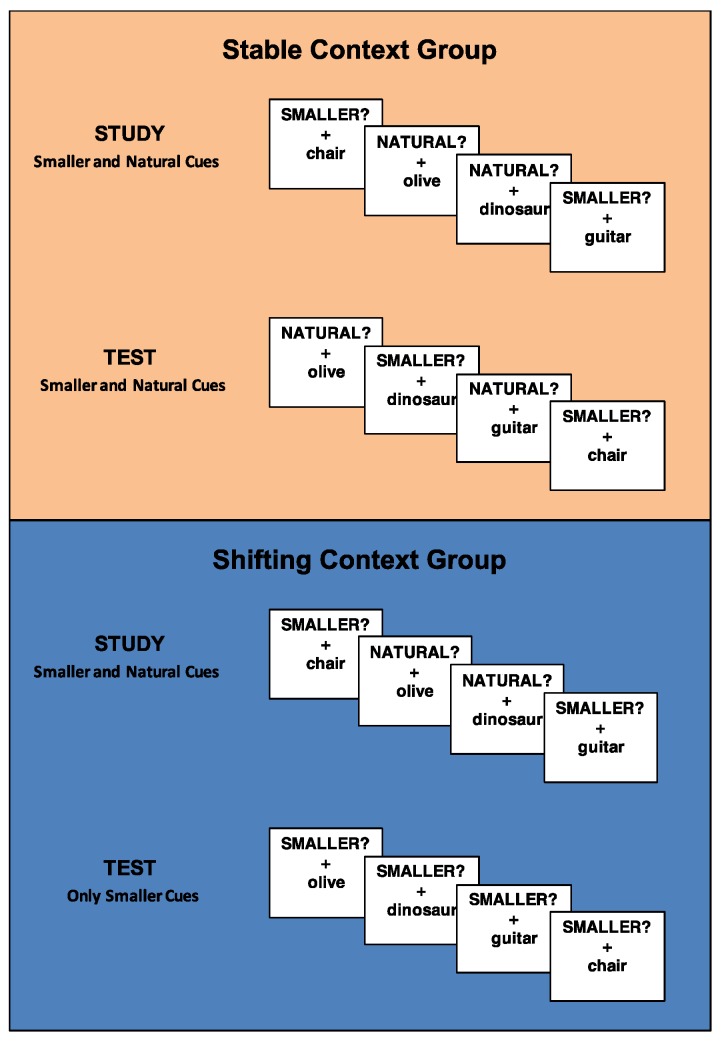
Task design for the stable context group and the shifting context group. Participants in the stable context group made two types of classification decisions (“SMALLER?” and “NATURAL?”) at study and test, whereas participants in the shifting context group made two types of classification decisions at study and only one type of classification decision (“SMALLER?”) at test.

During the study block, both groups of participants classified stimuli according to the “SMALLER?” and “NATURAL?” cues (mixed-task block). Importantly, during the test block, participants in the stable context group continued to make both classification decisions (i.e., mixed-task block → mixed-task block) while participants in the shifting context group switched to only classifying stimuli according to the “SMALLER?” cue (i.e., mixed task block → pure task block) (Figure 1). Thus, the global task set was manipulated between subjects and either remained consistent across blocks (stable context group) or changed across blocks (shifting context group).

**Figure 2:**
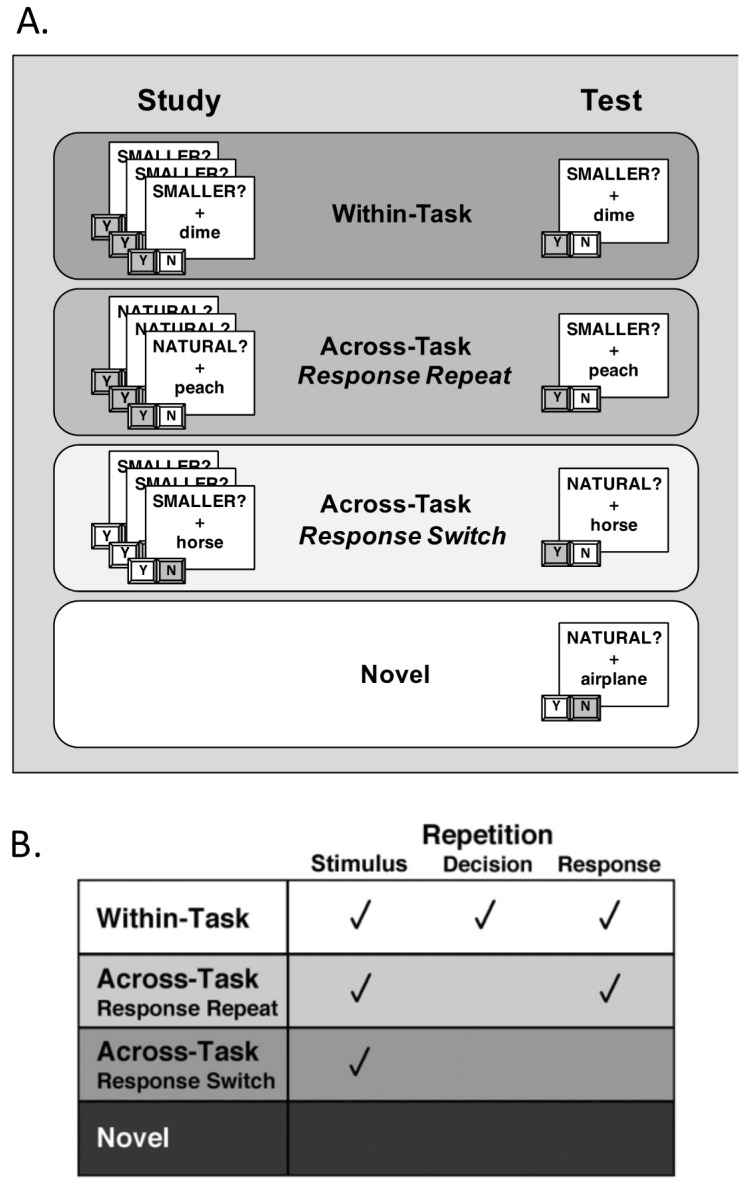
Task schematics showing levels of repetition. (**A**) During the study block, each primed stimulus was presented with the same decision cue three times, and participants pressed one of two buttons to indicate a “Yes” or “No” response. At test, primed stimuli were presented again, either with the same task cue (Within-Task trials) or with the alternate task cue (Across-Task trials). Of the Across-Task trials, half required the same response at test as at study (Across-Task Response-Repeat, AT-RR) and half required a different response (Across-Task Response-Switch, AT-RS). (**B**) The four test conditions differed according to repetition at the stimulus, decision, and response levels.

To manipulate S–R contributions to priming, the pairing of individual stimuli to individual decisions/responses was also manipulated across blocks within all participants (Figure 2). During the study block, participants encountered primed stimuli three times in a randomized order, intermixed with novel items. Half of these primed stimuli were classified under the size task and half were classified under the natural task. Importantly, each time a participant saw a repeated item at study, it was paired with the same “SMALLER?” or “NATURAL” task cue, thus requiring the participant to make the same “Yes” or “No” classification response (leading to the formation of S–R associations). During the test block, the primed stimuli from the study block were presented for a fourth time, again in a randomized order and intermixed with a second set of novel items. Importantly, here, the amount of repetition for the primed stimuli was manipulated by varying the relationship between the task cue paired with an item at study and the task cue paired with the same item at test (Figure 2A). For all participants, half of the primed stimuli were paired with the same task cue at test as at study (Within-Task), while half of the primed stimuli were paired with the alternate task cue at test (Across-Task). Of the across-task items, half required the same response at study and test (Response-Repeat), while half required a different response at test (Response-Switch). As a result of this manipulation, the amount of repetition between study and test blocks varied across conditions: (a) Within-Task (WT) trials contained repetition at the stimulus, decision, and response levels; (b) Across-Task Response-Repeat (AT-RR) trials contained repetition at the stimulus and response levels; (c) Across-Task Response-Switch (AT-RS) trials contained repetition only at the stimulus level; and (d) Novel trials served as baseline items that did not contain any amount of repetition (Figure 2B).

Thus, potential S–R contributions to priming were present in the two conditions in which responses repeated at test (WT and AT-RR trials [8]) and should be evident in faster reaction times when comparing these trials to Novel trials (e.g., WT and AT-RR priming). In addition, the study design allowed potential S–R contributions to priming to be isolated by directly comparing the magnitude of priming for AT-RR and AT-RS trials, as the only difference between these conditions was the validity of retrieved S–R associations. It is important to note that published data alternately suggest that the nature of the “response” representations that become associated with a stimulus during such semantic categorization priming tasks can be at the level of a particular action mapping (e.g., “left button press”) or a response label (e.g., “Yes”) (e.g., [4,5,9]). Though the present design does not distinguish between these two levels of response features, such “response” associations can be isolated from associations formed between stimuli and previously selected “decisions,” such as categorization decisions made about a stimulus prior to the initial selection of a response.

## 3. Results

Mean reaction time (RT) and accuracy data at test were determined for each condition and each group. Trials that were classified incorrectly at study or for which RTs were slower than two standard deviations from the mean were excluded (<2% of trials across participants). The overall pattern of behavior at test is reported in Table 1.

**Figure 3:**
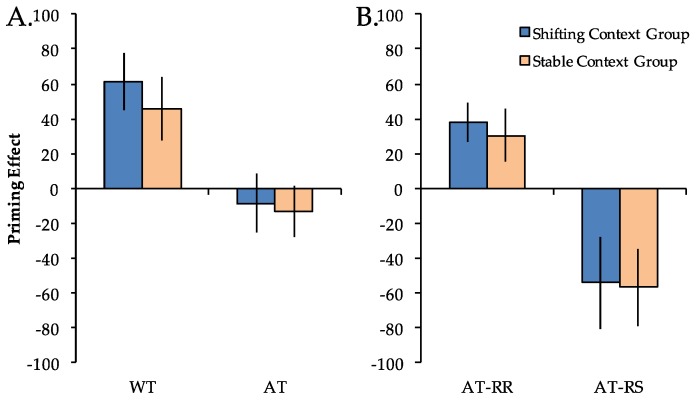
Priming effects (reaction time difference from Novel trials) in the shifting context group (blue) and the stable context group (orange). Priming scores were greater for Within-Task (WT) compared to Across-Task (AT) trials (**Panel A**) as well as for Across-Task Response Repeat (AT-RR) compared to Across-Task Response Switch (AT-RS) trials (**Panel B**), but did not differ between the stable and shifting context groups in any condition. Error bars indicate SEM.

To investigate the impact of stable vs. shifting global task contexts on priming, RT differences compared to Novel trials (priming scores) were first calculated for the WT and AT repetition conditions and entered into a 2 × 2 mixed model ANOVA with factors of group (stable context, shifting context) and repetition condition (WT, AT) (Figure 3A). Priming scores differed across repetition conditions (*F*(1, 38) = 39.62, *p* < 0.001), but did not differ across groups (*F*(1, 38) < 1). The main effect of repetition condition reflected significant positive priming in both groups for WT trials (*Fs*(1, 19) > 7.15, *ps* < 0.05) but not for AT trials (*Fs*(1, 19) < 1). Importantly, there was not a significant group x repetition condition interaction (*F*(1, 38) < 1). The pattern of results for the accuracy data mirrored that of the RT data, with a significant main effect of repetition condition (*F*(1, 38) = 12.12, *p* < 0.001), but no main effect of group (*F*(1, 38) = 2.76, *p* = 0.11) nor group x repetition condition interaction (*F*(1, 38) = 2.09, *p* = 0.16). An analysis of the pattern of results for the accuracy data did not demonstrate any evidence of a speed-accuracy tradeoff.

To more directly investigate S–R contributions to priming across groups, we next separated AT trials according to response type (AT-RR, AT-RS) and entered these priming scores into a 2 × 2 ANOVA (Figure 3B). There was a significant main effect of repetition condition (*F*(1, 38) = 30.27, *p* < 0.001) but no main effect of group (*F*(1, 38) < 1). The main effect of repetition condition reflected significant positive priming in both groups for AT-RR trials (*Fs*(1, 19) > 6.89, *ps* < 0.05) and significant negative priming in both groups for AT-RS trials (*Fs*(1, 19) > 4.56, *ps* < 0.05). Importantly, there was not a significant group x repetition condition interaction (*F*(1, 38) < 1). The pattern of results for the accuracy data mirrored that of the RT data, with a significant main effect of repetition condition (*F*(1, 38) = 52.40, *p* < 0.001), but no main effect of group (*F*(1, 38) = 1.36, *p* = 0.25) nor group x repetition condition interaction (*F*(1, 38) < 1). Again, an analysis of the pattern of results for the accuracy data did not demonstrate any evidence of a speed-accuracy tradeoff.

## 4. Discussion

The present findings provide important new insight into the automaticity and flexibility of S–R retrieval during priming. Prior behavioral and neuroimaging studies have suggested that S–R associations play a fundamental role in behavioral and neural priming [4,5,6,7,8,9,10]. However, the automaticity and flexibility of these learned associations is still a matter of debate. In the current study, we provide novel evidence that S–R associations can transfer across shifts in higher-level task contexts. Behavioral measures of priming revealed robust effects of S–R retrieval in both the stable and shifting context groups, evident in significant positive priming when responses repeated from study to test, even when tasks switched (AT-RR trials). The finding that S–R associations influenced priming regardless of whether the global task context at retrieval matched that at encoding informs our understanding of how cognitive and neural systems integrate the past and the present and supports a model of S–R learning in which learned S–R associations can transfer across changes in higher-level task states.

An outstanding question is why contextual factors such as global task set were not integrated into S–R memory traces during priming in the current study, particularly given prior evidence that visuomotor memory improves when the task set context (e.g., dual-task vs. single-task set) at encoding is reinstated at retrieval [15,16]. On the one hand, a lack of context-dependency would enable S–R associations to transfer to novel (but similar) contexts so that learning can generalize to new environments. On the other hand, this transfer across contexts could lead to behavior that is context-inappropriate (e.g., utilization behavior). This question is of particular interest given that it is known that other task representations (e.g., the decision cued by the task on a particular trial) are integrated with stimuli during repetition and affect priming [6,7,8,19,21]. It is possible that the transfer of S–R associations across task set contexts may depend on the degree of overlap in those task sets. In the current paradigm, the task set active at test in the shifting context group (a pure block of the “smaller” decision) partially overlapped with the task set that was active at study (a mixed block of “smaller” and “natural” decisions). This partial overlap in task sets may have encouraged S–R retrieval. If the shift had been to an entirely new task set—for example, a task set that oriented participants to features of the stimulus that were not activated at encoding—it is possible that the degree of S–R retrieval may have been reduced [18]. Future studies should investigate the extent to which other types of contextual shifts modulate S–R contributions to priming.

Another aspect of the current paradigm that merits future investigation is the difference between transferring to a task set in which two cognitive configurations are activated across trials (mixed task block) versus transferring to a task set in which only one cognitive configuration is activated across trials (pure task block). In the current paradigm, the stable task set group experienced mixed task blocks at both study and test, while the switching task set group experienced a mixed task block at study and a pure task block at test. One might have predicted that this difference in the task set context at test between groups might have impacted S–R retrieval and allowed for a greater S–R effect in the stable task set group, particularly if performing two tasks at test slowed operation time and allowed more time for stimulus-triggered retrieval processes [18]. However, mean RTs did not differ between groups, arguing against the availability of more operation time in the stable task set group, and indeed S–R contributions to priming did not differ between groups.

It is also important to note that our current paradigm cannot isolate the specific type of responses that become bound to stimuli in learned S–R associations and can transfer across contexts. For example, S–R learning in the current paradigm may reflect learned associations between stimuli and repeatedly-executed motor responses (e.g., left/right button presses). This would suggest that S–R associations at the level of motor output (or actions) may transfer across shifts in global task contexts, and leaves open the possibility that S–R associations at more abstract levels of response representation (e.g., the response label, ”Yes”) may not demonstrate such flexibility. Future studies should directly compare these different types of learned response associations to investigate whether the ability to transfer S–R associations across shifts in context depends on the nature of the response representation retrieved.

## 5. Conclusions

The finding that S–R associations can transfer across shifts in context emphasizes the automaticity and flexibility of S–R contributions to priming. However, the current results are limited by the fact that we only investigated one type of higher-order shift in context. Future work should investigate whether other types of contextual changes, such as changes in spatial or social contexts, modulate S–R retrieval [10]. Defining these boundary conditions will inform our understanding of how information in memory can be integrated with contextual information to guide goal-relevant behavior. Future work should also investigate the cognitive and neural mechanisms that enable us to use S–R associations in a context-free manner in some circumstances and in a context-dependent manner in others. Such work would inform neural models of S–R learning that aim to delineate the contributions of distinct brain regions, such as the hippocampus and frontal cortex, to repetition priming [4,8,9,21,22,23].

## Figures and Tables

**Table 1:** Reaction times (ms) and response accuracy varied across conditions (standard deviations in parentheses).

	Shifting Context		Shifting Context	
	RT	Accuracy	RT	Accuracy
**Novel**	880 (145)	0.88 (0.05)	951 (235)	0.83 (0.17)
**Within-Task**	813 (127)	0.80 (0.14)	903 (232)	0.84 (0.11)
**Across-Task**	888 (162)	0.77 (0.12)	964 (252)	0.78 (0.15)
Response-Repeat	840 (149)	0.86 (0.11)	921 (238)	0.87 (0.11)
Response-Switch	937 (182)	0.69 (0.15)	1008 (281)	0.70 (0.22)
